# *In vivo* rotational three-dimensional OCTA analysis of microaneurysms in the human diabetic retina

**DOI:** 10.1038/s41598-019-53357-1

**Published:** 2019-11-14

**Authors:** Enrico Borrelli, Riccardo Sacconi, Maria Brambati, Francesco Bandello, Giuseppe Querques

**Affiliations:** Department of Ophthalmology, University Vita-Salute, IRCCS Ospedale San Raffaele, Milan, Italy

**Keywords:** Retinal diseases, Diabetes complications

## Abstract

The aim of this study was to explore whether rotational three-dimensional (3D) visualization of optical coherence tomography angiography (OCTA) volume data may yield valuable information regarding diabetic retinal microaneurysm (MA) characteristics. In this retrospective, observational study, we collected data from 20 patients (20 eyes) with diabetic retinopathy. Subjects were imaged with the SS-OCTA system (PLEX Elite 9000, Carl Zeiss Meditec Inc., Dublin, CA, USA). The OCTA volume data were processed with a volume projection removal algorithm and then exported to imageJ in order to obtain a 3D visualization of the analyzed MAs. The rotational three-dimensional OCTA images were qualitatively and quantitatively investigated. A total of 52 MAs were included in the analysis. On rotational 3D OCTA images, the number of vessels associated with each MA varied between 1 and 4, and most MAs (59.6%) were associated with 2 vessels. Moreover, in 20 MAs (38.4%) these vessels seem to originate from the SCP, while 26 MAs (50.0%) had associated vessels originating from the DVC, and 6 MAs had associated vessels arising from both the SCP and DVC (11.6%). Most MAs (31/52) had a ‘saccular’ shape. The number of retinal layers occupied by each MA ranged between 1 and 3 and the inner nuclear layer was the retinal layer most frequently occupied by MAs. In conclusion, this study used an algorithm to obtain rotational three-dimensional visualization of retinal MAs. The MAs’ architecture is complex and 3D visualization may clarify the true vascular origin of these lesions, which is often mistaken using *en face* OCTA images.

## Introduction

Microaneurysms (MAs) are dilations of capillaries that usually appear as gross outpouchings of the vessel wall. While these vascular alterations can be found in different vascular beds (e.g. kidney, heart), they are mostly visualized and investigated in the retinal vessels of diabetic eyes^1^. Retinal MAs usually represent the earliest ophthalmoscopic feature appearing in eyes with diabetic retinopathy (DR)^2^.

Histopathologic attributes of MAs have been extensively studied in diabetic eyes^1,3,4^. In detail, postmortem analyses on DR eyes revealed that the majority of MAs originated in the inner nuclear layer (INL) and therefore in the deeper part of the retinal circulation^4^. Furthermore, MAs were demonstrated to have various shapes and three different morphologic patterns could be discerned: (i) saccular, (ii) fusiform, and (iii) focal bulges^4^.

Optical coherence tomography angiography (OCTA) is a relatively novel imaging technique that produces volumetric angiographic images by performing repeated OCT acquisitions in the same tissue location within a short time to detect scattering differences that relate to motion produced by blood flow in the retinal and choroidal microvasculature^5,6^. Using OCTA, the retinal vasculature may be visualized at different depths in order to display and assess three distinct retinal capillary layers: vessels located in the retinal nerve fiber and ganglion cell layers constitute the superficial retinal capillary plexus (SCP), while the middle capillary plexus and the deep retinal capillary plexus (DCP) are located at the inner and outer borders of the inner nuclear layer, respectively^7,8^. Because the middle and deep capillary plexuses consist of capillaries of uniform size and are separated by only a small distance, they are often grouped together as the deep vascular complex (DVC).

OCTA studies in diabetic patients have illustrated alterations in the retinal and choroidal perfusion^6,9–11^. Importantly, OCTA has granted the visualization and characterization of retinal MAs. In detail, OCTA analysis on eyes with DR demonstrated that MAs may be localized in all of the three capillary plexuses^12–14^. Notably, different OCTA studies confirmed previous histopathological evidences that MAs are mainly localized in the deeper retinal layers^12–14^. In addition, MAs’ characteristics were demonstrated to be relevant as prognostic factors for treatment response in eyes with diabetic macular edema (DME)^15,16^.

While OCT is considered a cross-sectional imaging modality, OCTA images are mainly displayed with *en face* visualization. The *en face* images may be obtained by segmenting the volumetric OCTA scan at specific depths. These volume sections defined by two topographic surfaces are called slabs. Using this strategy, the flow data within any slab are summed or projected into a two-dimensional *en face* image^5^. However, this two-dimensional (2D) visualization may offer limited knowledge of the relative frequency of the various types of microaneurysms and their origin, location and orientation within the retina. These shortcomings may be partially addressed with the development of three-dimensional (3D) illustrations, assuming that the acquired OCTA volume enables 3D visualization of the retinal and choroidal microvasculature. In addition, these 3D visualizations may be rotated on the 3 axes and this may further reduce limitations by overlapping anatomy and vessel foreshortening, as demonstrated for other imaging modalities (e.g. cerebral angiograms)^17^.

In this study we thus explored this technique in eyes with DR. To obtain this visualization we employed a partially novel process with a specifically designed algorithm to reduce volumetric projection artifact. Our aim was to understand whether the three-dimensional images generated by this technique yield valuable information regarding the configuration of retinal microaneurysms.

## Methods

### Study participants

This study is a retrospective, cross-sectional study. The authors in this study identified patients with diabetes and DR as determined by clinical examination^18^. The study was approved by the San Raffaele Institutional Review Board and adhered to the tenets of the Declaration of Helsinki and Health Insurance Portability and Accountability Act. Written informed consent was obtained from all subjects.

Exclusion criteria included any maculopathy secondary to causes other than DR (including presence of an epiretinal membrane or vitreomacular traction syndrome). Furthermore, we excluded poor quality images with a signal strength index lower than 6 (a measurement in a scale 0–10 indicating the level of retinal tissue signal with respect to the noise or background level in OCT data), as recommended by manufacturers.

All patients underwent a complete ophthalmological examination including best-corrected visual acuity (BCVA) assessment, slit lamp bio-microscopy and fundus examination by an experienced retina specialist.

### OCTA imaging

Patients underwent SS-OCTA imaging using the PLEX Elite 9000 device (Carl Zeiss Meditec Inc., Dublin, CA, USA) which uses a swept laser source with a central wavelength of 1050 nm (1000–1100 nm full bandwidth) and operates at 100,000 A-scans per second. This instrument employs a full-width at half-maximum (FWHM) axial resolution of approximately 5 μm in tissue, and a lateral resolution at the retinal surface estimated at approximately 14 μm. OCTA imaging of the macula included a 3 × 3-mm field of view area centered on the fovea (300 A-scans × 300 B-scans). this scan has a depth size of 3 mm.

### Image processing

To mitigate the influence of the projection effect on the three-dimensional visualization of microaneurysms, we used a prototype version of a volume projection removal algorithm. This algorithm is aimed at generating OCTA volumes without (or at least with less) projection artifacts. Different from slab-based artifact removal solutions, this algorithm proposes a solution to resolve artifacts within a volume instead of a single slab. In brief, the basic principle of this algorithm (available on the ARI network by Zeiss - https://arinetworkhub.com) consists on a set of serial consecutive steps in which a corrected volume is updated. At each step, a thin (small axial thickness) portion of the OCTA volume (target subvolume) is partially corrected and stored using a previously corrected portion of the OCTA volume located at inner positions. The target subvolume considered at each step is located at increasing depths in an overlapping manner. Using this process, each voxel within the OCTA volume is corrected in several consecutive steps.

**Figure 1:**
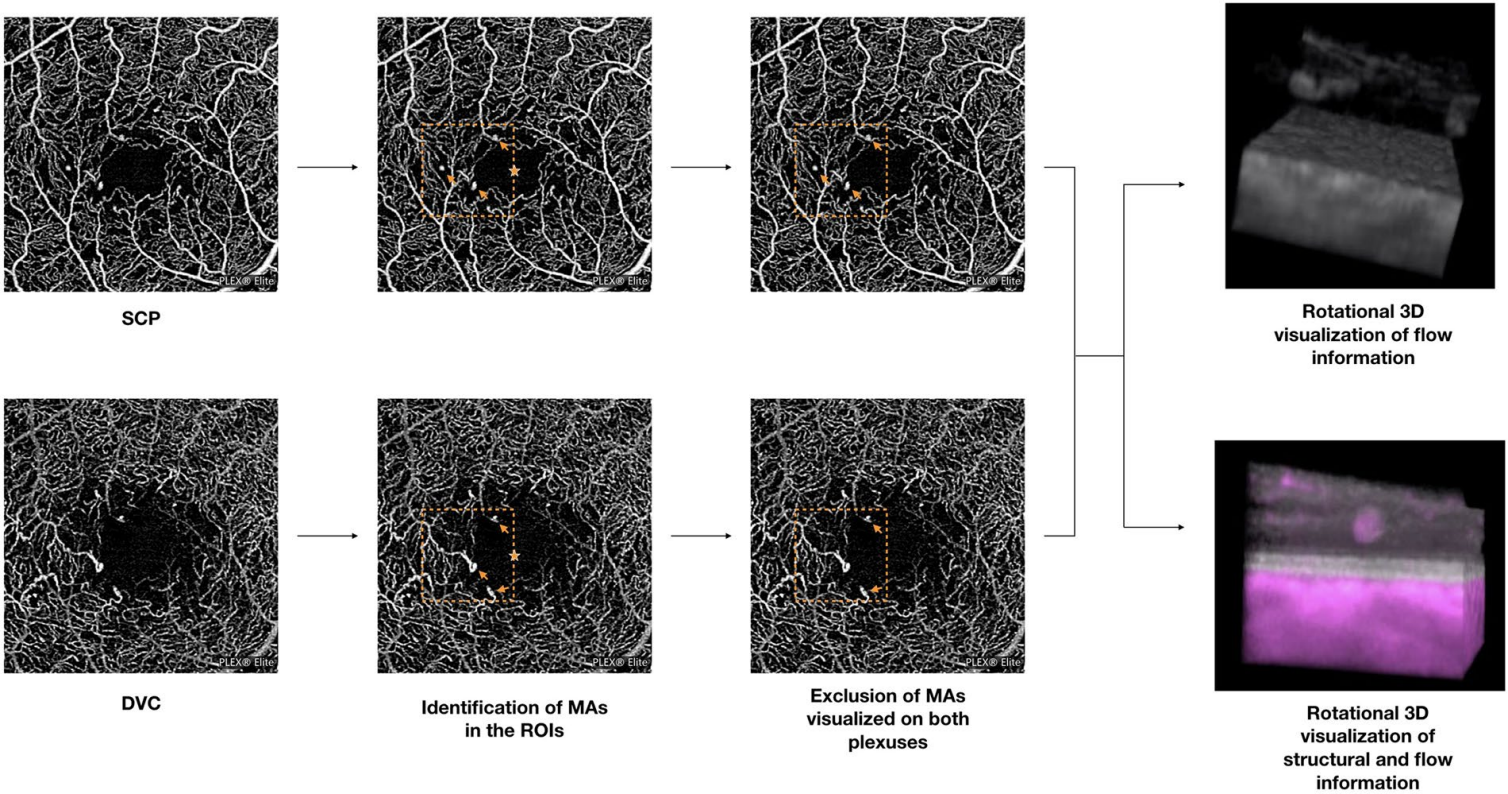
Representation of the algorithm used to process the images. The images of the superficial capillary plexus (SCP) and deep vascular complex (DVC) were graded for the presence of microaneurysms (MAs – indicated with arrows) in a 1-mm square region of interest (orange square) that was temporal and tangential to the foveal center (orange star). Those MAs visualized on both plexuses were included once. The OCTA volume scans were imported in ImageJ and elaborated (please see the “Methods” section for further details) in order to obtain the rotational 3D visualization of each MA, in which OCTA-based flow information is displayed as gray. Moreover, a second 3D visualization in which structural OCT and OCTA information had different colors (gray and magenta for structural and flow information, respectively) was used to topographically correlate MAs with retinal layers.

The obtained corrected OCTA volume scans were thus exported as uncompressed 8-bit raw-data file and imported in ImageJ software version 1.50 (National Institutes of Health, Bethesda, MD; available at http://rsb.info.nih.gov/ij/index.html)^19^ and re-oriented in space so that the first dimension correspond to the axial direction with increased pixel number as increasing depth, and the second and third dimensions correspond to the horizontal and vertical directions, respectively. Successively, before obtaining a three-dimensional visualization, an experienced grader (EB) identified MAs on the SCP and DVC *en face* OCTA images^12–14^. These MAs were identified in a 1-mm square region of interest (ROI) that was placed temporally and tangentially to the foveal center (Fig. 1). Importantly, those MAs detected on both SCP and DVC images were included once in the analysis and considered as visualized on both the SCP and DVC *en face* images in the analysis. Moreover, we excluded those MAs that were topographically located in correspondence of intraretinal cysts, assuming that they may alter the retinal architecture and thus significantly affect our topographical analysis. In addition, OCTA may display pseudoflow within intraretinal cysts^20^, this representing an additional reason to exclude these MAs. Finally, MAs were separately processed and investigated (Fig. 1). To do so, the “3D Viewer” plugin (downloadable on http://3dviewer.neurofly.de) was carried out to obtain the rotational 3D visualization of each selected MA (Fig. 1). This visualization was adopted to assess morphological characteristics of MAs.

**Figure 2:**
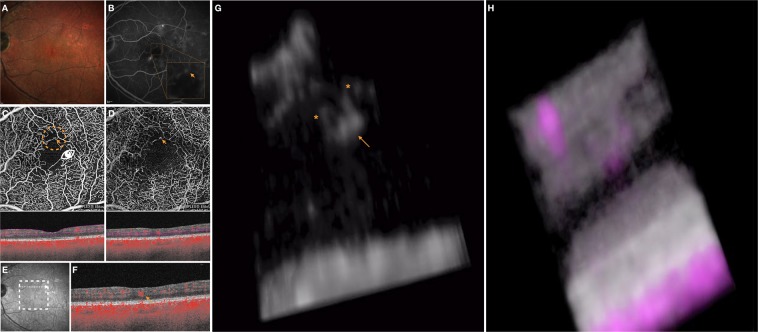
Multimodal imaging of the LE of a 61-year-old man diagnosed with non-proliferative diabetic retinopathy. The 3D OCTA visualization offers further details on the highlighted microaneurysm. (**A**) Multicolor image illustrates a few retinal hemorrhages and microaneurysms within the macula. (**B**) Fluorescein angiography (FA) shows several areas of pinpoint hyperfluorescence corresponding to the microaneurysms and some areas of blocked fluorescence due to the retinal hemorrhages. The orange arrow in the FA image indicates a MA which is evident on both the superficial capillary plexus (SCP) **(D)** and deep vascular complex (DVC) **(E)** 2D *en face* optical coherence tomography angiography (OCTA) images. Slabs used to visualize the *en face* OCTA images are shown. The white arrow in the near-infrared reflectance image **(E)** illustrates the location and direction of the OCTA B-scan **(F)** which shows the transversal visualization of the selected MA within the retinal layers. The 3D OCTA visualization (image (**G**) is the frame obtained by visualizing the circular region of interest highlighted on image (**C**) and visualized from the angle marked with the white eye) displayed the selected MA (highlighted with the orange arrow) and two vessels (highlighted with the orange asterisks) associated. The 3D visualization of combined OCTA (magenta) and structural OCT (gray) information **(H)** demonstrates that the MA is mainly contained in the INL and OPL.

**Figure 3:**
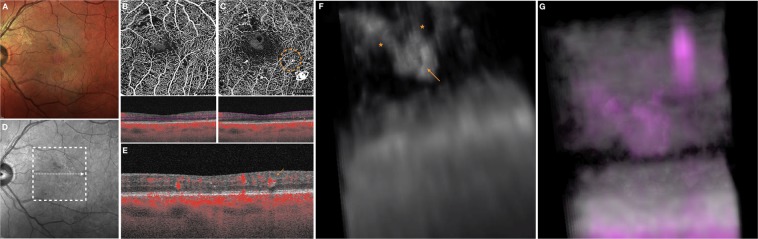
Multimodal imaging of the LE of a 67-year-old man diagnosed with non-proliferative diabetic retinopathy. A deep microaneurysm may be visualized in 3D and this improves the understanding of its shape, orientation and localization within the retinal layers. (**A**) Multicolor image demonstrated the presence of retinal hemorrhages and microaneurysms (MAs) within the macula. **(B)** The superficial capillary plexus (SCP) and **(C)** deep vascular complex (DVC) 2D *en face* optical coherence tomography angiography (OCTA) images illustrate the presence of several MAs. Segmentation to obtain these two *en face* images is also displayed. The white arrow in the near-infrared reflectance image **(D)** illustrates the location and direction of the OCTA B-scan **(E)** which shows the transversal visualization of the MA indicated with the orange arrow in the DVC image. The 3D OCTA visualization **(F)** displayed the selected MA (highlighted with the orange arrow) and two vessels (highlighted with the orange asterisks) associated. For completeness, image **F** is the frame obtained by visualizing the circular region of interest highlighted on image **C** and visualized from the angle marked with the white eye. The 3D visualization of combined OCTA (magenta) and structural OCT (gray) information **(H)** demonstrates that the MA is mainly contained in the INL, OPL and ONL.

In order to study the relationship between structural OCT and OCTA-detected flow, structural OCT volume and OCTA volume data were merged. Therefore, the “3D Viewer” plugin was carried out to obtain a second 3D visualization in which structural OCT and OCTA information had different colors (magenta and gray for flow and structural information, respectively, Figs 1–3, Videos 1, 2). The latter visualization was used to topographically correlate MAs and structural OCT information.

### Grading and statistical analysis

The three-dimensional OCTA and OCT findings were carefully examined by an experienced grader (EB).

Image grading included:

- Number of vessels associated with each MA and their apparent origin (SCP or DVC).
- The localization of each MA in the neuroretinal layers, which include the ganglion cell complex (GCC) that represents the combination of the ganglion cell (GCL) and inner plexiform layer (IPL), the INL, the outer plexiform layer (OPL), and the outer nuclear layer (ONL).
- The shape of each MA. In detail, MAs were categorized as previously described^4^, as follows: (i) ‘saccular’ if the dilation was asymmetric around the long axis of the associated vessel(s); (ii) ‘fusiform’ if the dilation was symmetric around the long axis of the associated vessel(s) or (iii)‘focal bulge’ if too small and irregularly shaped to be classified as (ii) or (iii).
- Orientation of each MA, in relation to retinal structures, retinal pigment epithelium (RPE) and Bruch’s membrane. In detail, MAs were classified as (i) parallel to RPE or (ii) oblique to RPE.
- The size of MAs, measured on the longer axis they develop.

In order to investigate the interobserver agreement, 10 randomly sampled microaneurysms were also graded by a second reader and Cohen’s kappa was calculated. The analysis included descriptive statistics (using Microsoft Office Excel software; version 14.0, 2010, Redmond, WA) for demographics and main clinical data, and qualitative and quantitative descriptions of the imaging characteristics. Spearman’s correlation coefficient was tested to evaluate the linear correlation among 3D OCTA variables.

## Results

A total of 20 eyes with DR from 20 diabetic patients were included in the analysis. Table 1 summarizes characteristics of enrolled patients and analyzed eyes.

**Table 1:** Characteristics of diabetic patients.

**Number of eyes enrolled (patients)**	20 (20)
**Age**, years, mean ± SD	59.3 ± 14.9
**Gender**	
- M, n (%)	15 (75%)
- F, n (%)	5 (25%)
**BCVA**, Snellen equivalent, mean	20/25
**Level of DR**, n	
- Mild	3
- Moderate	11
- Severe	3
- Proliferative	3
**Previous treatment**, n	
- Focal/grid laser	2
- Panretinal laser	6
- Anti-VEGF	9
- Dexamethasone implant	2
- Treatment naïve	5

**M:** males; **F:** females; **n:** number of eyes; **BCVA:** best corrected visual acuity; **VEGF:** vascular endothelial growth factor.

A total of 63 microaneurysms were identified on the 2D OCTA images (22/63 [34.9%] on the SCP *en face* image and 41/63 [65.1%] on the DVC *en face* image, respectively). Of these, 11 MAs were present on both SCP and DCP images and were thus included once in the analysis. Therefore, a final number of 52 MAs were visualized with a 3D rotational approach and graded (Table 2).

**Table 2:** Characteristics of analyzed microaneurysms.

	Number of MAs (percentage)
**Total number of analyzed MAs**	52 (100%)
**Number of vessels associated with each MA**	
- 1 vessel	16 (30.8%)
- 2 vessels	31 (59.6%)
- 3 vessels	4 (7.7%)
- 4 vessels	1 (1.9%)
- 5 vessels	0 (0.0%)
**Origin of the vessels associated with each MA**	
- Only SCP	20 (38.4%)
- Only DVC	26 (50.0%)
- Both SCP and DVC	6 (11.6%)
**Retinal layers occupied by each MA**	
- GCC	23 (44.2%)
- INL	42 (80.8%)
- OPL	31 (59.6%)
- ONL	4 (7.7%)
**Shape**	
- Saccular	31 (59.6%)
- Fusiform	8 (15.4%)
- Focal bulge	13 (25.0%)

**MA:** microaneurysm; **SCP:** superficial capillary plexus; **DVC:** deep vascular complex; **GCC:** ganglion cell complex; **INL:** inner nuclear layer; **OPL:** outer plexiform layer; **ONL:** outer nuclear layer.

### Grading of 3D rotational images displaying flow information

On rotational 3D OCTA images, the number of vessels associated with each microaneurysm varied between 1 and 4 (mean = 1.8) and most MAs (59.6%) were associated with 2 vessels. Moreover, in 20 MAs (38.4%) these vessels seem to originate from the SCP, while 26 MAs (50.0%) had associated vessels originating from the DVC, and 6 MAs had associated vessels arising from both the SCP and DVC (11.6%). Importantly, 5 out of 52 MAs identified on the 2D DVC *en face* images (and NOT on the SCP *en face* images) were actually connected with vessels originating in the SCP (without any apparent connection with the DVC). On the contrary, no MA recognized on the 2D SCP *en face* images (and NOT on the DVC *en face* images) were connected only with vessels originating from the DVC (Table 2).

Considering the whole cohort of identified MAs, the majority, 31, were ‘saccular’, 13 were ‘focal bulges’, while only 8 were ‘fusiform’. Median size of MAs was 137.7 microns (Table 2).

### Grading of 3D rotational images displaying flow and structural information

The number of retinal layers occupied by each MA ranged between 1 and 3. The INL was the retinal layer most frequently occupied by MAs. In detail, the GCC layer was occupied by 23 (44.2%) MAs, the INL had 42 (80.8%) MAs crossing, the OPL was invaded by 31 (59.6%) MAs, and 4 (7.7%) MAs were also accommodated in the ONL (Table 2).

Regarding the orientation of MAs, 17 MAs were parallel to the RPE, while 35 MAs were oblique on the three plans along their route throughout the retinal layers. Fourteen of 17 MAs parallel to the RPE were located within the GCC or OPL.

Spearman correlation analysis found significant positive correlation between MA’s dimension and number of retinal layers crossed by each MA (Rho = 0.321 and p = 0.041). No significant correlations were found between the number of vessels connected with each MA and both MA’s dimension and number of retinal layers crossed (Rho = 0.018 and p = 0.909, Rho = −0.059 and p = 0.716, respectively).

### Interobserver agreement

Cohen’s kappa was 1.0 in all the qualitative assessments.

## Discussion

In this retrospective, cross-sectional study we applied a novel algorithm to volumetric OCTA data in order to obtain rotational three-dimensional visualizations of microaneurysms in eyes with diabetic retinopathy. Overall, we observed that this approach may be effective to display these vascular alterations and may offer new important insight into the characterization of these lesions.

Data from a number of studies using distinct approaches clarified the origin and characteristics of MAs. Histopathologic characteristics of MAs have been extensively studied in postmortem analyses on DR eyes. In 1999, Moore *et al*.^4^ employed an immunohistochemical technique in conjunction with confocal laser scanning microscopy to study diabetic eyes without altering the tissue architecture. The authors demonstrated that the majority of MAs was associated with just 2 vessels. Moreover, the latter study defined three microaneurysm morphologies (saccular, fusiform, and focal bulges). Of note, the latter study’s results showed that the majority of MAs originated in the INL, although they were also found to originate throughout the thickness of the retina from the border of the ONL and OPL to the GCC. Although structural OCT provides limited anatomic information regarding the retinal vascular layers, Wang and colleagues^21^ nicely assessed ultrastructural characteristics of MAs in diabetic patients. Interestingly, the authors demonstrated that most MAs spanned more than one retinal layer, this finding further suggesting a complex distribution of these vascular abnormalities^21^. By adding adaptive optics to OCT imaging, Karst *et al*.^22^ were recently able to improve the transverse resolution of structural OCT and to further confirm these findings.

Taken together, these studies suggest that, even though 2D *en face* OCTA images were demonstrated to be helpful for displaying MAs in diabetic eyes^12–16^, this visualization might be at least limited in offering a comprehensive characterization of MAs.

We add to the literature by reporting the rotational 3D visualizations of MAs in eyes with DR. This approach was revealed to be useful for the identification and characterization of these vascular malformations. Our results confirmed previous histopathological findings^4^, as we demonstrated that most of analyzed MAs were associated with 2 vessels, suggesting no tendency to develop at vascular junctions. Furthermore, even with this small cohort, we were able to provide an imaging evidence that three morphologic patterns could be easily discerned and that the “saccular” pattern represents the most common configuration.

One of the most notable observations from our study was that the majority of microaneurysms occupied the INL, although they were also found to expand throughout the thickness of the retina, as most MAs occupied at least two retinal layers. These results confirm previous histopathological and imaging reports, as explained above^4,21,22^. Given that a single MA may reside in different retinal layers, the latter observation suggests that the same MA might be visualized on two distinct 2D *en face* OCTA images using two different OCTA segmentations (e.g. SCP and DVC), and thus erroneously counted twice, as correctly suggested in a previous report^12^ and confirmed in our study. Furthermore, importantly, although most MAs are located in the INL, where the DVC is accommodated, some MAs seem to represent dilations of vessels arising from the SCP rather than the DVC, which may additionally reflect the extent of the vascular deviation from normal architecture in diabetic eyes. Importantly, a few MAs resulted connected with both SCP and DVC vessels, the latter aspect suggesting that these vascular abnormalities might also affect vessels connecting large-caliber arterioles and venules in the SCP to small vessels located in the DVC, these connecting vessels fully characterized in healthy subjects using OCTA^23,24^.

Notably, in our study cohort we observed a median size of MAs of 137.7 microns. In a previous important histopathological report on MAs, the authors demonstrated that MAs range in size between 14 and 136 microns^4^, this suggesting that our measures tend to overestimate the actual MA size. However, discordances between OCTA and histology measurements were already demonstrated and the OCTA overestimation seems to be secondary to a limited resolution of this technique^25^.

Of note, the 3D image technique provides a way to assess the orientation of these lesions with respect to the retinal layers, RPE and Bruch’s membrane. In detail, each MA appeared to have a particular orientation on the three dimensions and most MAs have an oblique orientation (forming angles >0 with the three axes of the three-dimensional Cartesian coordinate system) moving toward the outer retinal layers. Assuming that most MAs develop throughout different retinal layers, we speculate that this orientation might be secondary to the presence of Müller cells, whose processes are known to have an oblique orientation within the macula. Notably, we showed that those MAs with a horizontal configuration mainly occupy the GCC or OPL, this probably reflecting the presence of horizonal cells in these layers.

Our study has some limitations which should be considered when assessing our findings. First, the sample size of the cohort is relatively small. However, this represents a pilot study using 3D visualization to describe diabetic MA and future independent replicative studies will be essential to validate our findings. Moreover, our study cohort displayed heterogeneity in previous treatments, which may have further undermined our analysis. Another limitation of our study is the employment of a single time point for each patient. A prospective longitudinal evaluation of diabetic patients will shed further light on the role of the three-dimensional approach in the follow-up of these lesions. Furthermore, it should be considered that OCTA images must be interpreted with caution owing to a variety of artifacts^26^. Most importantly, projection artifacts might potentially cause alterations in the OCTA assessment (both 2D and 3D). Although we used a novel volume project artifact removal algorithm to lighten this issue and data on file produced by the company (Carl Zeiss Meditec Inc., Dublin, CA, USA) supports the efficacy of this algorithm to reduce projection artifacts in a 3D visualization, this algorithm could not be quantitatively validated by comparing it with other algorithms used in other devices and projection artifacts might have still partially altered our analysis. Finally, another limitation is intrinsic to the OCTA technique, which is not able to distinguish the absence of flow from that under the slowest detectable flow. Assuming this, we are not able to undoubtedly assert that analyzed MAs are not associated with more vessels than those displayed. Finally, we selected only those MAs in a region of interest tangential to the fovea, where the foveal avascular zone is shaped by the retinal plexuses merging at its edge^27^. This have undoubtedly increased the rate of MAs visualized on both the SCP and DVC *en face* images. However, the OCTA scan edge may be characterized by a reduced image quality^26^ and thus we felt the safest strategy was to exclude this edge from the analysis. Finally, those microaneurysms displayed on both SCP and DVC *en face* images were included once in the 3D analysis.

In conclusion, this study used a novel algorithm to obtain rotational three-dimensional visualization of microaneurysms in diabetic eyes. This approach was demonstrated to be effective in displaying these vascular alterations and to be useful to offer new insight into MAs’ pathogenesis and characteristic. Assuming that 2D *en face* OCTA images are limited by overlapping anatomy and vessel foreshortening, our results suggest that 3D images may be more appropriate to show MAs by resolving these limitations. Future studies with longitudinal follow up may provide further insight into the three-dimensional aspects of MAs and clarify their role in the prognosis of patients with diabetic retinopathy.

## Supplementary information

VIDEO LEGENDS

Video 1. Rotational 3D visualizations of the diabetic microaneurysm illustrated in Figure 2.

Video 2. Rotational 3D visualizations of the diabetic microaneurysm illustrate in Figure 3.

## Data Availability

The data used to support the findings of this study are available from the corresponding author upon request.
