# Supplementary material for: In vivo rotational three-dimensional OCTA analysis of microaneurysms in the human diabetic retina
Source: Sci Rep. 2019 Nov 14;9:16789. doi: 10.1038/s41598-019-53357-1 (PMC6856183; doi:10.1038/s41598-019-53357-1)
Supplement: Supplementary file 1 — VIDEO LEGENDS [file 41598_2019_53357_MOESM1_ESM.pdf]

In vivo rotational three-dimensional OCTA analysis of microaneurysms in the human diabetic retina

Enrico Borrelli, MD, FEBO;[1] Riccardo Sacconi, MD, FEBO;[1] Maria Brambati, MD;[1] Francesco Bandello, MD, FEBO;[1] and Giuseppe Querques, MD, PhD.[1]

[1] Department of Ophthalmology, University Vita-Salute, IRCCS Ospedale San Raffaele, Milan, Italy.

VIDEO LEGENDS

Video 1. Rotational 3D visualizations of the diabetic microaneurysm illustrated in Figure 2. In the first part of this video (flow information is illustrated in grey color) it is possible to appreciate the microaneurysm rotating, which improves the visualization of its shape, orientation and connections with retinal vessels. Two vessels seem to be associated with this microaneurysm. In the second part of the video, the flow information (magenta) cohabits with structural information (gray), which allows to identify the localization of the microaneurysm within the retinal layers.

Video 2. Rotational 3D visualizations of the diabetic microaneurysm illustrate in Figure 3. In the first part of this video showing flow information the microaneurysm rotates and two associated vessels are present. In the second part of the video, the microaneurysm (magenta) was demonstrated to be mainly contained in the INL, OPL and ONL
